# Cardiovascular Disease Risk Modeling for Astronauts: Making the Leap From Earth to Space

**DOI:** 10.3389/fcvm.2022.873597

**Published:** 2022-05-19

**Authors:** Janice L. Huff, Ianik Plante, Steve R. Blattnig, Ryan B. Norman, Mark P. Little, Amit Khera, Lisa C. Simonsen, Zarana S. Patel

**Affiliations:** ^1^National Aeronautics and Space Administration, Langley Research Center, Hampton, VA, United States; ^2^KBR, Houston, TX, United States; ^3^National Aeronautics and Space Administration, Johnson Space Center, Houston, TX, United States; ^4^Division of Cancer Epidemiology and Genetics, Department of Health and Human Services (DHHS), Radiation Epidemiology Branch, National Cancer Institute, National Institutes of Health (NIH), Bethesda, MD, United States; ^5^Division of Cardiology, University of Texas Southwestern Medical Center, Dallas, TX, United States; ^6^National Aeronautics and Space Administration, NASA Headquarters, Washington, DC, United States

**Keywords:** clinical prediction model, radiation-induced cardiovascular disease, space radiation, risk modeling, astronaut, biomarker, individual risk, radiation epidemiology

## Abstract

NASA has recently completed several long-duration missions to the International Space Station and is solidifying plans to return to the Moon, with an eye toward Mars and beyond. As NASA pushes the boundaries of human space exploration, the hazards of spaceflight, including space radiation, levy an increasing burden on astronaut health and performance. The cardiovascular system may be especially vulnerable due to the combined impacts of space radiation exposure, lack of gravity, and other spaceflight hazards. On Earth, the risk for cardiovascular disease (CVD) following moderate to high radiation doses is well-established from clinical, environmental, and occupational exposures (largely from gamma- and x-rays). Less is known about CVD risks associated with high-energy charged ions found in space and increasingly used in radiotherapy applications on Earth, making this a critical area of investigation for occupational radiation protection. Assessing CVD risk is complicated by its multifactorial nature, where an individual's risk is strongly influenced by factors such as family history, blood pressure, and lipid profiles. These known risk factors provide the basis for development of a variety of clinical risk prediction models (CPMs) that inform the likelihood of medical outcomes over a defined period. These tools improve clinical decision-making, personalize care, and support primary prevention of CVD. They may also be useful for individualizing risk estimates for CVD following radiation exposure both in the clinic and in space. In this review, we summarize unique aspects of radiation risk assessment for astronauts, and we evaluate the most widely used CVD CPMs for their use in NASA radiation risk assessment applications. We describe a comprehensive dual-use risk assessment framework that supports both clinical care and operational management of space radiation health risks using quantitative metrics. This approach is a first step in using personalized medicine for radiation risk assessment to support safe and productive spaceflight and long-term quality of life for NASA astronauts.

## Introduction

In recent years, NASA has pursued long-duration missions to the International Space Station and is solidifying plans to return to the Moon. With the renewed capability to launch astronauts from United States soil, NASA will continue to expand the boundaries of human exploration with prospective missions to Mars and beyond. As plans for these ambitious missions develop, the need to manage the health risks associated with spaceflight becomes increasingly important. NASA identifies five main categories of spaceflight hazards: microgravity, isolation and confinement, distance from Earth, hostile and closed environments, and exposure to space radiation. Health risks escalate as missions increase in duration and extend beyond Earth's protective magnetosphere. For radiation specifically, these risks include solid cancers and leukemia, in-mission and late neurocognitive and neurobehavioral decrements, acute radiation syndrome, degenerative conditions related to accelerated aging, immune dysfunction, and late occurring radiation-induced cardiovascular disease (RICVD), including ischemic heart disease and stroke (1).

Increased morbidity and mortality associated with RICVD is a concern for radiation protection on Earth and in space. The risks to the heart and circulatory system, mainly ischemic heart disease and stroke, are well-established from Earth-based clinical, environmental, and occupational studies of high dose gamma- and x-ray exposures (above ~3 Gy) (2–6). Positive correlations with risk are also observed at much lower doses. Establishing a threshold below which no excess disease occurs remains uncertain; however, multiple studies show significant risks at absorbed doses of 0.5 Gy and lower (7–10). For radiation protection, the International Commission on Radiological Protection recommends a practical threshold of 0.5 Gy, below which effects would be observed with probability of 1% or less. This is in the dose range expected for exploratory missions to the Moon and Mars (Table 1) (2, 11, 12). Given that it is also quite likely that the heavy ions found in the space environment are more damaging than gamma- and x-rays for these outcomes, there is a reasonable concern that space doses below 0.5 Gy could induce non-negligible RICVD risks. Consequently, there is a major interest in characterizing heart and circulatory risks associated with space radiation exposure and the combined stressors of spaceflight (13).

**Table 1:** Dose estimates for radiation exposures on earth and in space (2, 11, 12).

Radiation exposure scenario^*^	**Dose (mGy)**
International Space Station (1 year)	60–120
Lunar Surface Mission (42 days total)	25
Sustained Lunar Operations (1 year)	100–120
Deep-Space Habitat (1 year)	175–220
Mars Mission (650 to 920 days)	300–450
Chest X-ray	0.1–0.23
Computed Tomography-Chest	20–30
Computed Tomography-Full Body	50–100
Cardiac Catheterization	12–40
Mammogram	0.6–2.9
RICVD Threshold Dose (ICRP)	500

* *All space-based dose estimates are average rates external to the body inside spacecraft with 20 g/cm^2^ of aluminum shielding during solar minimum conditions. ICRP, International Congress on Radiation Protection*.

Currently, the NASA radiation risk assessment model predicts risk of exposure-induced death (REID) due to radiogenic cancers as the most likely cause of increased mortality due to space radiation exposures received by astronauts (14). This model uses United States population disease rates as the baseline for calculating risk and performs scaling to account for excess risk due to radiation exposure received during spaceflight. For future long-duration missions where higher radiation exposures will cross over the estimated threshold for RICVD, approaches to incorporate CVD mortality risk into the NASA radiation risk model are important for a more inclusive risk assessment (15). There is also the potential for additional risk from the other spaceflight hazards, such as microgravity and hostile and closed environments. As quantitative data for these hazards becomes available, expansion of risk modeling capabilities to include these combined or synergistic risks will be important for a comprehensive risk assessment framework (16).

Heart disease is the number one cause of death in the United States, and many types of cardiovascular disease (CVD) are readily detectable in the clinic (17). Numerous options exist for CVD prevention and management, including enhanced surveillance, lifestyle modifications, and medications, which if employed early, can prevent disease development and adverse outcomes (17, 18). Since astronauts are at elevated risk due to the radiation exposures received during spaceflight, it is important that primary prevention approaches and risk communication strategies used on Earth are adapted to accommodate the unique risks associated with spaceflight.

The development of risk assessment models and tools for RICVD is complicated by the multifactorial nature and complex spectrum of cardiovascular responses to radiation exposure. Non-radiation risk factors such as lifestyle and genetics strongly influence individual risk and may significantly impact risk estimates, especially at lower radiation doses. One way to overcome these limitations is to use baseline risk estimates from subpopulations with risk profiles representative of an astronaut population. Subpopulation risk estimates can be derived from CVD clinical prediction models (CPMs), which evaluate specific disease outcomes and are widely used for primary prevention of CVD (18–20). These models calculate stratified risk estimates based on individual factors such as age, sex, family history, and traditional CVD risk factors. A broad range of these tools are available that focus on multiple CVD disease endpoints and risk covariates. Two models in particular, Astro-CHARM (21) and a multimodality risk prediction tool described by de Lemos et al. (22), were developed with specific consideration for the needs of NASA astronauts. In this review, we describe an approach for extending the NASA radiation risk assessment model to provide individual RICVD risk estimates using input from CPMs. This extension provides a dual-use capability that will support the needs of both mission planners as well as clinical care of the astronauts at the individual level (23).

## Cardiovascular Health in Astronauts

**Figure 1:**
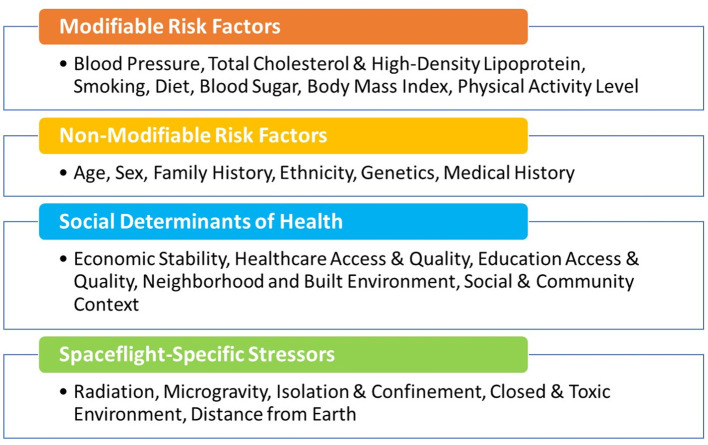
Modifiable and non-modifiable cardiovascular risk factors, including social determinants of health (25) and those unique to astronauts during space missions.

Protecting cardiovascular health of the astronauts before, during, and after spaceflight is vital for mission success and to ensure long-term quality of life. NASA astronauts undergo careful cardiovascular health monitoring starting in their early days as astronaut candidates, throughout their careers, and into retirement. Preventive medical examinations that include cardiovascular heath assessments are performed on an annual basis as part of the Lifetime Surveillance of Astronaut Health, an occupational surveillance program for both current and former astronauts (24). These regular screenings include the typical core measurements and assessment of the modifiable risk factors noted in Figure 1 and additional non-traditional risk factors or biomarkers such as coronary artery calcium scores. As a group, the NASA astronauts are at low risk for CVD based on their modifiable risk factors, but individual risk also depends on non-modifiable risk factors such as age, sex, and family history (Figure 1). Astronauts generally have healthy lifestyles with a high level of physical fitness, good dietary practices, and normal weight. Therefore, the overall CVD incidence and mortality in the United States astronaut corps is below that of the general United States population (26), but it is important to note that there is still variability in risk between individual astronauts.

NASA maintains an active research program to characterize how the spaceflight environment impacts human health, which includes studies focused on the impacts of altered gravity on the cardiovascular system. Complex physiological changes occur when the human body experiences microgravity. Some of these adaptations may quickly resolve upon return to gravity, but the possibility for adverse impacts to long-term health have also been suggested. In particular, cardiovascular deconditioning is seen in the astronauts in the short-term and may also have lasting implications (16, 27, 28). This includes fluid shifts as blood volume is redistributed from the lower to the upper body, total blood volume reductions, and alterations in nominal flow patterns and pressure gradients (29, 30). These adaptations have been shown to contribute to orthostatic intolerance and functional impairment in cardiac output and aerobic capacity (31).

**Figure 2:**
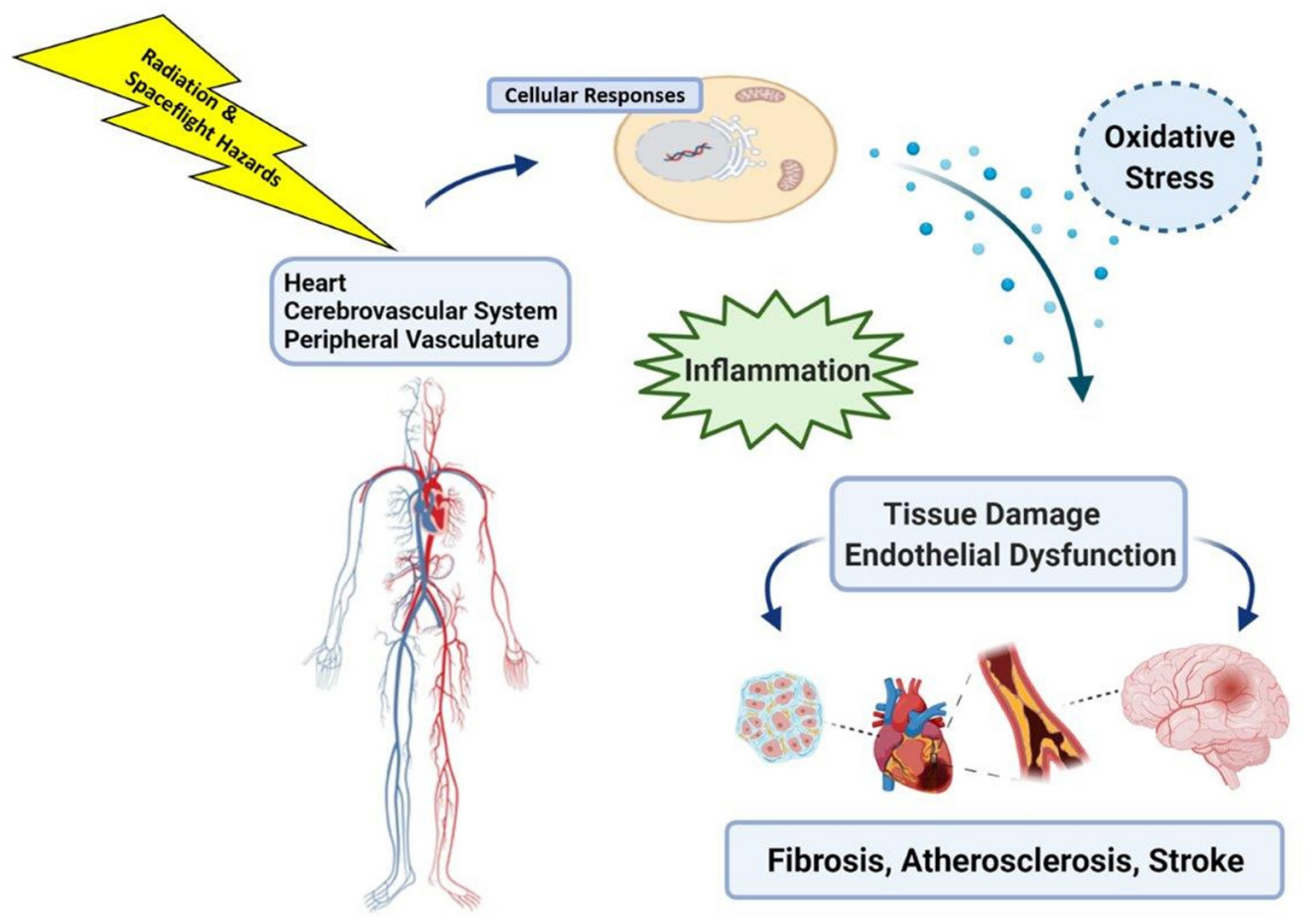
Space radiation, and exposure to other hazards of spaceflight such as microgravity, can induce cell and tissue level changes that include oxidative stress and inflammatory responses in the heart and vasculature that may impact long-term cardiovascular health. Image created with Biorender.com.

Ongoing investigations have also included assessment of a variety of biochemical markers of oxidative stress and inflammation such as high-sensitivity C-reactive protein, and assessment of atherosclerotic indices such as carotid intima-media thickness and brachial artery flow-mediated dilation (32). Because early events in RICVD also include oxidative stress and inflammation that lead to tissue damage, it is plausible that the mechanisms that underpin CVD risks from spaceflight (Figure 2) may be shared across multiple spaceflight hazards (29, 33). This raises concern about the potential for cardiovascular decrements that may combine or synergize during and after spaceflight (16, 29). Characterizing cardiovascular risks from these non-radiation spaceflight hazards, and assessing the potential for interaction with space radiation, are important considerations and can be addressed in NASA risk models in the future as data characterizing the spectrum and magnitude of these risks becomes available.

## Radiation Environment in Space

During spaceflight, astronauts are exposed to particle radiation from galactic cosmic rays (GCR), solar particle events, and trapped radiation from the Van Allen Belts (34). Compared to the gamma- and x-ray radiation responsible for most of the exposures on Earth, GCR have unique characteristics in terms of both their physical nature and the amount of biological damage they cause. GCR include high-linear energy transfer particle radiation that produces densely ionized tracks as it traverses cells and tissue. This contrasts with gamma- and x-rays, which are low-linear energy transfer and sparsely ionizing. Particle radiation can create complex molecular damage, with persistent oxidative stress and clustered DNA double-strand breaks that are difficult to repair (35). The complex damage is associated with the higher efficiency of these high-energy ions for producing detrimental biological outcomes (34, 36). Studies in animal models of high energy charged particle exposure, similar to certain types of GCR, have shown cardiovascular effects at doses lower than those required to cause cardiovascular changes if low-LET radiation is used (37). In particular, exposure of male C57BL/6N mice to 1 GeV protons or 1 GeV/n ^56^Fe ions induced cardiac infiltration of CD68^+^ cells (monocytes and macrophages), increased DNA oxidation, myocardial fibrosis, and modified cardiac function, both at baseline and in response to induced myocardial infarction (38–40). Exposure of male CBA/CaJ mice to 300 MeV/n ^28^Si ions caused prolonged apoptosis and increased expression of the common pro-inflammatory cytokines interleukin (IL)-1β, IL-6, or tumor necrosis factor-α in the heart (41). Low doses of high energy 1 GeV or 600 GeV/n ^56^Fe ions have been shown to cause long term alterations in DNA methylation in various organ systems *in vivo* and *in vitro* (42–44).

## Current Management of Space Radiation Health Risks by NASA

NASA's decision process for the management of space radiation health risks relies, in part, on permissible exposure limits that are set by the Office of the Chief Medical Officer and are defined in the NASA Standard 3001, Volume 1 (45). This document covers cancer and non-cancer risks and specifies short-term, yearly, and career limits. Exposure monitoring is done using onboard vehicle and personal dosimetry, and predictive models are used to estimate the associated REID. By controlling exposures and health impacts, the limits play an important role in operational risk mitigation and are used in vehicle design, mission planning, and research (11, 13, 46).

REID is a metric of radiation risk that is calculated over the course of a lifetime, covering the period from the age of exposure until an assumed maximum attained lifespan. It is one of several commonly used metrics of excess lifetime risks from an exposure, and it specifically quantifies the increase in cause-specific deaths attributable to the exposure. The NASA Space Cancer Risk model was developed to assess REID and is specifically tailored to the space environment (14, 47). The model uses approaches similar to those used by the National Institute of Health to assess human health risks from occupational, accidental, and medical radiation exposures on Earth (48, 49).

**Figure 3:**
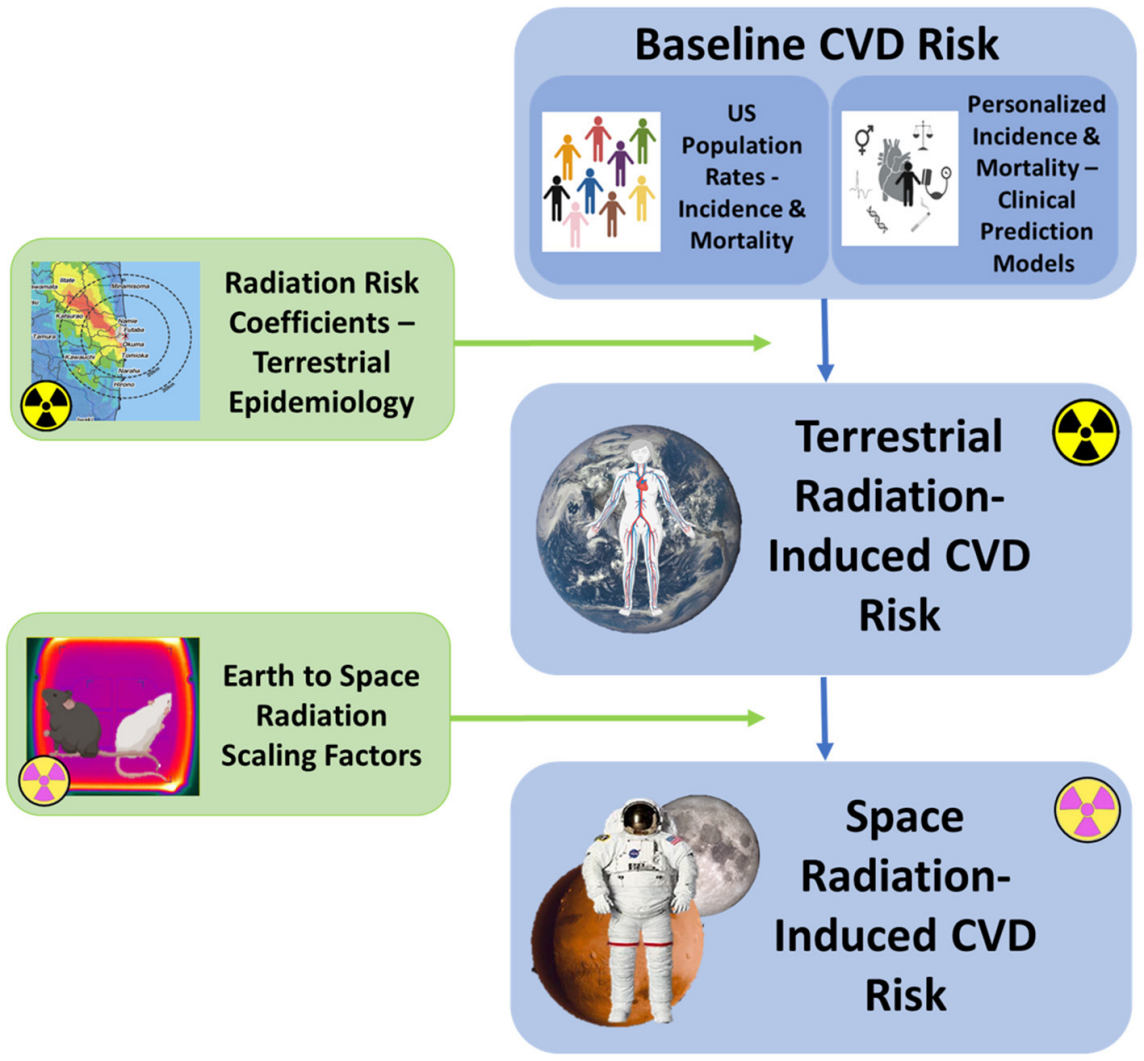
Schematic overview of the major components of the NASA REID model proposed for space radiation CVD risk estimation. Risk calculations contain three components, the same as described for the NASA Space Cancer Risk model (14). The first component involves identification of the baseline CVD incidence and mortality rates, derived either from the United States population or from an integrated clinical prediction model (CPM) for individualized risk estimation. The second component calculates the excess RICVD risk from Earth-based exposures. The third component performs the calculations required to extrapolate this risk to the spaceflight environment using radiation scaling factors that account for the potentially more biologically damaging nature of space radiation. Scaling factors for CVD endpoints are currently under investigation (15).

An overall framework for calculation of risks for space radiation-induced CVD based on the current NASA REID model is illustrated in Figure 3. The model consists of three main components: an assessment of baseline disease risk, a calculation of excess radiation-induced disease risk in terrestrial populations, and an application of space-radiation specific scaling factors to calculate REID in astronauts. The approach used for REID for disease mortality can also be applied to disease incidence by defining the quantity risk of exposure-induced cases (REIC), where the initial population-based mortality rate in the equation for REID calculations is replaced by an incidence rate (50). Ideally, this type of risk framework will have capability to calculate risks of CVD incidence and mortality over relevant time periods to meet both clinical and operational needs.

### Calculation of Baseline CVD Risk

Extension of the NASA REID model to incorporate additional risks due to RICVD was first demonstrated in a preliminary study by Cucinotta et al. (15), where a lifetime model incorporating combined mortality due to radiation-associated cancer and CVD was described. This model uses background mortality rates for CVD from the average United States population as published by the Center for Diseases Control and Prevention. As noted earlier, astronauts are considered to be healthier than the general United States population, even when accounting for smoking status and healthy weight. There is also individual variability within the astronaut cohort that will impact risk estimates. Therefore, an alternative approach is to use individual baseline CVD risk estimates derived from a clinical prediction model (CPM) as the starting point for calculation of excess risk due to radiation exposure during spaceflight.

### Assessment of Excess RICVD Risk

The second component of the NASA REID model incorporates excess risk coefficients that are derived from human populations exposed to terrestrial radiation. Excess risk per unit radiation dose can be expressed as either an excess relative risk or an excess absolute risk, where the former assumes the radiation risk is proportional to, and the latter assumes it is independent of, the background disease rates. An excess relative risk model (without excess absolute risk) will be used for calculation of excess risk for RICVD for initial computational efforts. In Cucinotta et al. (15), the excess relative risk used in risk calculations was obtained from a meta-analysis of human epidemiological data from the Life Span Study of atomic bomb cohorts and other environmental and occupational workers exposed to uniform, whole-body low-LET radiation (51). The REID calculations, which assume population with given overall mortality and baseline CVD rates as a function of age and sex, are entirely standard, and as described by UNSCEAR (52). The baseline CVD rates used could be derived directly from certain non-smoking populations (53), alternatively by scaling rates from a standard US population *via* the known ratio of CVD rates in astronauts and a general US population (54).

Excess relative risk values obtained from a meta-analysis of available epidemiological data for circulatory diseases following low to medium levels of radiation exposure are presented in Table 2 (7). The values are given by disease categories and include confidence intervals that were obtained by assuming a linear no-threshold dose response and no age dependence. These values provide an estimate of the magnitude of the risk for subcategories of CVD at exposure levels that are within the range of planned long-duration exploration missions like a 3-year Mars mission (~0.5 Gy). These or similar estimates (55) can be used in development of the dual-use RICVD model described in this paper. Besides radiation dose, typical variables that are also considered include age, sex, age at exposure, and other environmental and lifestyle factors (e.g., smoking). Accordingly, the estimation of disease risk from radiation exposure is derived from large-scale human population terrestrial studies. Using terrestrial cohort studies is a necessary approach because it is not practical to directly estimate increased disease risks associated with space radiation exposure from spaceflight data due to the small population size of the astronaut corps and the limited power of the studies (56).

**Table 2:** Excess relative risk coefficients for CVD (7).

**Disease**	**Coefficient (Gy^**−1**^)**	**95% confidence interval**
Ischemic heart disease (ICD-10: I20-I25)	0.082	(0.057–0.106)
Ischemic heart disease, low dose-rate	0.147	(0.087–0.207)
Non-ischemic heart disease (ICD-10: I26-I52)	0.094	(0.078–0.111)
Cerebrovascular disease (ICD-10: I60-I69)	0.236	(0.062–0.410)
Cerebrovascular disease, low dose-rate	0.308	(0.075–0.542)
Other circulatory diseases (ICD-10: I00-I19, I53-I59, I70-I99)	0.137	(0.049–0.322)

*ICD, International Classification of Disease*.

### Scaling RICVD Risk for Space Radiation Exposures

The third component of the NASA REID model incorporates scaling factors for space radiation. These factors quantitatively account for differences in the type and magnitude of biological effects of space radiation compared to gamma- and x-rays (called radiation quality factors and relative biological effectiveness factors). They are also used to account for differences that result from the chronic nature of exposure in space (called dose and dose-rate effectiveness factors). Scaling factors are largely derived from *in vitro* and *in vivo* radiobiology data obtained at ground-based experimental facilities such as the NASA Space Radiation Laboratory at Brookhaven National Laboratory. Here, space radiation exposures can be simulated, and the unique biology associated with particle radiation can be studied using experimental model systems (11). The use of scaling factors provides a translational path by which the knowledge from the large-scale, terrestrial human cohorts can be extrapolated to estimate the risks to astronauts from space radiation exposure, thereby enabling risk assessment approaches to make the leap from Earth to space. In the RICVD risk model described by Cucinotta et al. (15), scaling was performed using relative biological effectiveness factors, and, since values specific for endpoints related to CVD are still under investigation, estimates for the blood forming organs were used as a surrogate (57, 58). No scaling for dose or dose-rate was included (2, 15, 59).

## Clinical Prediction Models for CVD and Unique NASA Needs

### Clinical Prediction Models

The concept of cardiovascular risk factors was first introduced by the Framingham Heart Study investigators William Kannel and Thomas Dawber in their 1961 landmark paper “Factors of Risk in the Development of Coronary Heart Disease-Six-Year Follow-up Experience” (60). They showed that high blood cholesterol levels, elevated blood pressure, smoking, and electrocardiographic abnormalities were associated with an increased risk of incident coronary heart disease over a 6-year follow-up of the Framingham cohort. After over 50 years of research, their findings have been confirmed by numerous studies, and many other modifiable (diabetes, sedentary lifestyle, diet, and psychosocial) and non-modifiable (age, sex/gender, family history, and ethnicity/race, etc.) factors. Additional CVD risk factors have since been identified (Figure 1) (61). For NASA astronauts, the distinctive hazards of spaceflight further expand this list, and because spaceflight hazards are harder to control, their presence increases the impetus for early monitoring and intervention in this unique group of individuals.

Identification of the common, modifiable CVD risk factors enabled the development of population-based and personal interventions that significantly contributed to the decline in CVD mortality in the last several decades (62). The population-based approach focuses on health promotion activities and actions that influence the environment (i.e., physical, social, economic, and regulatory). Individual approaches focus on high-risk or affected individuals through direct interventions (17). Today, with the increasing availability of clinical data and biomedical knowledge, there is an ever-expanding list of cardiovascular CPMs. These models predict atherosclerotic CVD (ASCVD) and global CVD risk inclusive of a broader array of cardiovascular outcomes or individual disease components such as risk of stroke.

**Figure 4:**
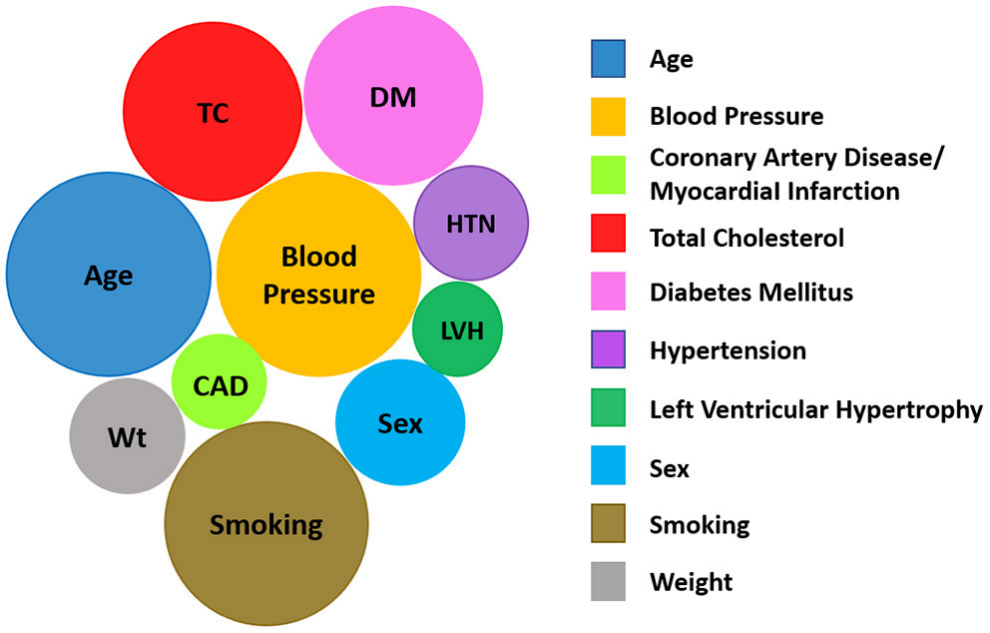
The 10 most common variables included in CPMs predicting the development of incident CVD based on data in the Tufts Predictive Analytics and Comparative Effectiveness Clinical Prediction Model Registry (63). The size of a given circle reflects the number of models that consider that variable. (Image modified from Tufts PACE CPM Data Visualization, available at http://pacecpmregistry.org/data-visualization/index-conditions-top-variables/).

CPMs for use in the general population have been reviewed (63–65). In particular, Wessler et al. (63) describes the Tufts Predictive Analytics and Comparative Effectiveness Center CPM registry (through 2015) with information on model characteristics, performance, covariates, and predicted outcomes. Figure 4, derived using the Tufts database, shows that age, blood pressure, cholesterol, diabetes, and smoking, i.e., the traditional CVD risk factors, are the most commonly used covariates in CPMs that are built to predict the development of incident CVD in the general population.

A few important limitations exist for the use of a CPM in the NASA risk model. In general, many models are not externally validated (66). Additionally, the accuracy of CPMs can be limited and even the best risk models achieve a C-statistic or C-index of only ~0.80. This means that a 20% probability still exists that the CPM is not able to discriminate an individual within the study population likely to develop CVD on follow-up from one less likely to do so (67). Also, many models are not generalizable, or reproducible, when used on a population unrelated to the development population (68). Lastly, CVD risk assessment on the individual level still lacks precision. Baseline CVD CPMs do not provide the user with uncertainty estimates and therefore may not reflect the within-person uncertainty in risk (69).

Regardless, CVD is the leading cause of death worldwide, and it is widely recognized that early intervention and control of the modifiable risk factors offers the best approach to decrease disease burden and overall mortality. Therefore, the use of CPMs provides a valuable initial step to aid in stratification of patients and promotes personalized care so that the most at-risk individuals are identified. Because CPMs are widely used for this purpose, they are well-suited for inclusion into the NASA radiation models for individualized astronaut risk assessment.

It should be emphasized that the NASA REID model is not currently used in clinical practice. The goal of this work is to lay the groundwork to develop something similar to current clinical guidelines for primary prevention that use CVD risk models to inform certain decisions. The first step is to incorporate more individualized assessments that include additional risk from spaceflight exposures.

### Requirements of an Ideal CPM for NASA Radiation Risk Assessment

The formulation of risk assessment tools to support decision makers and clinicians will help to ensure optimal health and quality of life for individual astronauts. Most CVD CPMs yield a 10-year risk score because they are intended to identify individuals at high risk that would most benefit from an early primary preventive treatment (70). However, this work does not necessarily address lower risk cohorts, like the astronauts, where longer term risks (>30 years) may be more relevant (70). Furthermore, in order to estimate shorter-term risks relevant to mission planning and integrate the CVD risk in the NASA radiation risk framework, the risk as a function of age, rather than integrated over a 10- or 30-year period will be needed. A suitable CPM needs to be extended that can calculate rates in yearly bins for integration into the NASA risk model.

Many standard risk prediction models do not show appreciable risks until ages 40+. While many astronauts do not have their first mission until ages over 40, the standard methods of calculating REID (52) will estimate risks even for comparatively young ages.

The characteristics or attributes identified for an ideal CPM for use in development of an individualized CVD risk prediction model for NASA astronauts are as follows: (1) outputs are endpoints consistent with those measured by RICVD epidemiological studies; (2) calculates risk for asymptomatic, healthy, middle-aged and older adults from US populations to enhance generalizability; (3) includes the ability to calculate lifetime incidence and mortality on a yearly basis for time frames extending to 30 years or longer; (4) incorporates predictive biomarkers as covariates to augment precision; (5) incorporates standard clinical interventions such as lifestyle modification or blood pressure with ability to predict effects of interventions on individual risk profiles over time; and (6) outputs include uncertainty estimates. This list of features serves as a starting point for identification of a suitable CPM for radiation risk modeling.

For the first attribute, model output, the outcomes of interest should be based on current knowledge of RICVD. We can assume that these are the broad outcomes aligning with the International Classification of Disease (ICD) codes identified in Table 2 and can be adequately summarized as outcomes related to the major adverse cardiac and cerebrovascular events.

The second attribute considers demographic characteristics of the development population and how well these align with those of the NASA astronaut cohort. Models based on relatively healthy populations that are in the age range of astronauts are preferred. Because astronauts maintain a healthier lifestyle, the timeframe (latency) for CVD development may be long (several decades). Therefore, it would also be useful to calculate an astronaut's CVD risk for time horizons extending to 30 years (third attribute), in addition to the standard 10-year timeframe.

A fourth attribute is the incorporation of CVD biomarkers to more accurately assess RICVD risk. Any biomarkers considered for inclusion should be established as a reliable predictor of increased CVD risk in humans and useful for screening in asymptomatic individuals. For example, subclinical disease imaging for coronary artery calcium can identify early development of atherosclerosis and is currently used at NASA (21, 32). Also, useful biomarkers will be suitable for evaluation in preclinical models, which will provide a translational path that allows biomarker validation in ground-based experiments using simulated space radiation exposures. For clinical applications, the biomarkers should pass the test of the three fundamental questions outlined by Morrow and de Lemos (71): (1) “Can the clinician measure the biomarker?”, (2) “Does the biomarker add new information?”, and (3) “Does the biomarker help the clinician to manage patients?” A large number of new biomarkers have been considered for inclusion in CVD CPMs, as evidenced by the extensive literature (20, 72).

The fifth attribute is the ability to evaluate impact of interventions such as blood pressure or lipid lowering agents. This is important due to the widespread clinical use of these agents, and it is also important to be able to estimate treatment effects and see impact on individual risk for clinical decision-making (73). It should be noted that there are many lifestyle changes that can lower CVD risk in a population (for example lipid lowering drugs such as statins). However, such interventions do not prevent radiation-induced CVD, simply lower the baseline risk in the population. Additional knowledge is needed on mechanisms and interactions of radiation with the other cardiovascular risk factors in order to identify potential countermeasures and estimate efficacy.

Finally, the NASA risk model considers a variety of uncertainties related to transfer of risks across populations, the radiation risk coefficients, the use of scaling factors, and statistical uncertainties. The ability to account for uncertainties on the risk estimates derived from the CPMs will be an important consideration in the future.

## CVD Clinical Prediction Models for NASA Astronauts

There have been several robust and extensive reviews of CPMs (63–65). However, there are unique requirements needed for use in NASA radiation risk modeling. With those in mind, various models were considered for their relevance and are summarized in Table 3. Models include those currently incorporated into clinical guidelines in both the US and Europe (87). Evaluation was based on attributes defined above and for predictive performance and degree of validation, risk factors, outcome measures and time horizons, inclusion of biomarkers, and the degree of similarity of the cohort used for model development to the astronaut population. Two specific models, Astro-CHARM and a multimodality risk prediction tool described by de Lemos et al. (22), were developed specifically with consideration for NASA applications and are described in more detail below. Descriptions for the other models are available in the Supplementary Material.

**Table 3:** Summary of clinical prediction models evaluated for use in NASA radiation-induced cardiovascular disease risk modeling.

**CPM**	**Traditional risk factors**	**Other factors and biomarkers**	**Population**	**Age (yrs)**	**Time horizon and outcome**	**Comments**
Astro-CHARM (21)	Age, Sex, DM, Sm, SBP, RxBP, TC, HDL	FHx, hs-CRP, CAC	US; Multi-ethnic	40–65	10-yr composite ASCVD	Family history; multiple biomarkers (CAC); medications; demographics-multi-ethnic, contemporary US population; older ages
de Lemos et al. (22)	Age, Sex, DM, Sm, SBP, RxBP, TC, HDL	Eth, hs-CRP, CAC, hs-cTnT, NT-ProBNP, LVH	US; Multi-ethnic	45–84	10-yr composite score (CVD death, MI, stroke, coronary or peripheral revascularization, IHF, or AF) and ASCVD	Multiple biomarkers (CAC); medications; multi-ethnic, contemporary US population; older ages
Framingham risk score (70, 74)	Age, Sex, DM, Sm, SBP, RxBP, TC, HDL	NA	US; Caucasian	30–75	10-yr risk of ASCVD (coronary death, nonfatal MI, and fatal or nonfatal stroke) and others; 30-yr risk of hard events (coronary death, MI, stroke)	Multiple outcomes; several model versions; ethnicity not considered; no biomarkers; older data
ACC/AHA pooled cohort equation (75)	Age, Sex, DM, Sm, SBP, RxBP, TC, HDL	Race	US; Caucasian, African American	20–79	10-yr risk of first ASCVD event (coronary death, nonfatal MI, and fatal or nonfatal stroke); 30-yr and lifetime risk	30-yr and lifetime risk: no family history or biomarkers; ACC/AHA clinical practice guidelines
MESA risk calculator (76)	Age, Sex, DM, Sm, SBP, RxBP, TC, HDL	BMI, Race, use of statins, FHx, CAC	US; Multi-ethnic	45–84	10-yr risk of hard CHD events (MI, resuscitated cardiac arrest, fatal CHD and revascularization)	Family history; biomarker (CAC); multi-ethnic, contemporary US population; older ages
Reynolds risk score (77, 78)	Age, Sex, DM, Sm, SBP TC, HDL	PHx <60 yrs, hs-CRP	US; Caucasian	>45	10-yr risk ASCVD and coronary revascularization	Parental history; biomarker (hsCRP); largely Caucasian, ethnicity not considered; older ages
INTERHEART modifiable risk score (61, 79)	Age, Sex, DM, Sm, SBP	APO B/A, diet, physical activity, psychosocial stress	52 countries; Multi-ethnic	All ages	Incident MI	Based on global, multi-ethnic data; biomarker (APO B/A); psychosocial stress; incidence MI only
SCORE/2 (80, 81)	Age, Sex, Sm, SBP, TC, HDL	NA	European populations	40–69	10-yr risk of fatal and non-fatal CVD (CHD or stroke)	European regional models; relative risk for younger pop; cardiovascular risk age; no biomarkers; SCORE2 includes non-fatal endpoints
QRISK 2/3 (82–84)	Age, Sex, DM, Sm, SBP, RxBP, TC, HDL	Eth, FHx, BMI, KD, AF, RA, Psy, Mig, SLE, SMI, CS	UK; Multi-ethnic	35–74	QRISK2: 1 to 15-yr risk of CVD; Lifetime risk of CVD, CAD, MI, stroke, TIA QRISK3: 10-yr risk of CVD events; relative risk; heart age	Lifetime risk; multiple covariates; family history of premature heart disease in first degree relative <60 yrs of age; heart age; no biomarkers; calibrated for UK
LIFE-CVD (85, 86)	Age, Sex, DM, Sm, SBP, RxBP, non-HDL	BMI, FHx, aspirin and lipid therapy	US; Multi-ethnic	45–80	10-yr, lifetime risk, CVD-free life expectancy, ASCVD, treatment effects	Contemporary US population; risk estimates with 1-yr age intervals; family history, treatment benefit predictions; no biomarkers; older ages

*ACC/AHA, American College of Cardiology/American Heart Association; AF, atrial fibrillation; ASCVD, atherosclerotic cardiovascular disease; BMI, body mass index; CAC, coronary artery calcium; CAD, coronary artery disease; CHD, coronary heart disease; CHF, congestive heart failure; CS, corticosteroids; CVD, cardiovascular disease; DM, diabetes mellitus; Eth, ethnicity; FHx, family history; HDL, high-density lipoprotein; hs-CRP, high-sensitivity C-reactive protein; hs-cTnT, high-sensitivity cardiac troponin T; IHF, incident heart failure; KD, kidney disease; LVH, left ventricular hypertrophy; MI, myocardial infarction; Mig, migraine; NA, not applicable; NT-ProBNP, N-terminal pro-B-type natriuretic peptide; PHx, parental history of MI <60 yrs of age; Psy, psycho-social factors; RA, rheumatoid arthritis; RxBP, blood pressure treatment; SLE, systemic lupus erythematosus; Sm, smoking; SBP, systolic blood pressure; SMI, serious mental illness; TC, total cholesterol; TIA, transient ischemic attack; TG, triglycerides; yr, year; UK, United Kingdom; US, United States*.

### Astro-CHARM

The Astronaut Cardiovascular Health and Risk Modification (Astro-CHARM) model (21) was developed with the goal of performing personalized risk assessments for NASA astronauts. This CPM is an integrated ASCVD risk calculator that targets middle-aged adults with no previous history of ASCVD, and as such, aligns well with the astronaut cohort. The model was developed using patient data from the Dallas Heart Study (1,491 subjects), Multi-Ethnic Study of Atherosclerosis (4,029 subjects), and Prospective Army Coronary Calcium Project (1,862 subjects) cohorts. The follow-up of patients was 10.9 years, during which 304 ASCVD events (fatal and non-fatal myocardial infarction or stroke) were recorded. The covariates in this Cox proportional hazards model included traditional risk factors (age, sex, ethnicity, total cholesterol, high-density lipoprotein cholesterol, systolic blood pressure, hypertension medication, smoking, diabetes) as well as coronary artery calcium, high-sensitivity C-reactive protein, and family history of myocardial infarction in first degree relatives at any age. Inclusion of these biomarkers and family history provides improved prediction for 10-year risks for ASCVD outcomes. There are two versions of the models to allow for flexibility depending on whether high-sensitivity C-reactive protein has been measured. Astro-CHARM was externally validated in the Framingham Heart Study cohort (2,057 subjects) where it demonstrated good discrimination for ASCVD (C-statistic of 0.78 and 0.79 for full and no C-reactive protein versions, respectively).

### Multimodality Risk Prediction Tool

The developers of Astro-CHARM also published a second, extended CPM: a multimodal risk prediction tool that incorporates biomarkers reflective of multiple CVD pathways, i.e., N-terminal pro-B-type natriuretic peptide and high-sensitivity cardiac troponin T, in addition to traditional risk factors to assess 3-year (88) and 10-year risk for global CVD (22). This study includes participants from the Dallas Heart Study (2,202 subjects) and Multi-Ethnic Study of Atherosclerosis (6,621 subjects) cohorts. The primary outcome evaluated was the time to the first global CVD event comprised of cardiovascular death, myocardial infarction, stroke, coronary or peripheral revascularization >3 months after enrollment, incident heart failure, or atrial fibrillation. Other outcomes are hard ASCVD event endpoints, including fatal or nonfatal myocardial infarction and fatal or nonfatal stroke, and coronary heart disease (fatal or nonfatal myocardial infarction), incident heart failure, all-cause mortality, and CVD mortality.

Baseline traditional risk factor parameters included age, sex, race/ethnicity, smoking status, diabetes, total cholesterol, high-density lipoprotein cholesterol, systolic blood pressure, blood pressure medication, and statin use, as well as creatinine and body mass index depending on the outcome of interest. Five biomarkers were added to these base models-left ventricular hypertrophy by electrocardiogram, coronary artery calcium, N-terminal pro-B-type natriuretic peptide, high-sensitivity cardiac troponin T, and high-sensitivity C-reactive protein-resulting in an improvement of the C-statistic from 0.74 to 0.79. Among these biomarkers, coronary artery calcium demonstrated the largest hazard ratio for ASCVD and coronary heart disease events while N-terminal pro-B-type natriuretic peptide and high-sensitivity cardiac troponin T had the largest hazard ratios for all cause and CVD mortality and heart failure. With the test results combined in a simple integer score (0 to 5 points), a >20-fold gradient in risk for primary global CVD outcome after >10 years of follow-up was observed for the individuals with the highest risk score compared with those with the lowest scores. A similar multimodality strategy was used to develop a short-term (3-year) global CVD model, which may be a useful strategy for preflight screening (88).

## Discussion

Over the last several decades, strategies for primary prevention of CVD have proven to be very effective in lowering overall risk in human populations on Earth. With this in mind, a framework to begin adapting these strategies to include the increased risks associated with space radiation exposures is presented. Attributes of an ideal CPM for use in assessing RICVD risk in the astronaut corps were identified, and the most widely used CVD CPMs were evaluated, emphasizing these criteria. All the models reviewed had outputs relevant for evaluation of RICVD, meeting the first outlined requirement. While none of these models met all criteria, Astro-CHARM and the de Lemos multimodality risk model (22) provided the most consistent match largely due to the coverage of a broad range of CVD outcomes, addition of biomarkers, and focus on middle-aged healthy adults from an ethnically diverse United States-based study population. Nevertheless, further optimization of these and/or other models is needed to more closely match the requirements outlined for an ideal model for NASA risk assessment. For example, identifying cohorts with similar demographic and physical characteristics to astronauts is challenging as illustrated by a comparison of demographics and risk factors between the Astro-CHARM and multimodal risk tool cohorts, and astronauts and cosmonauts, shown in Table 4. This indicates that calibration and validation for NASA applications needs to be carefully planned. Also, both the Astro-CHARM and de Lemos multimodal CPMs estimate risk for a 10-year time frame, and therefore further work would be needed to extend these to longer time frames (up to 30 years) that are required for NASA radiation risk assessment. Other options are to develop an approach using a series of predictive models, like that used in the new ASCVD risk estimator plus calculator (90). The LIFE-CVD model was noteworthy for the ability to output longer-term risk estimates, which is required for healthier populations, and importantly for the capability to estimate impact of standard therapeutic interventions. The assessment of psychosocial stress as a risk factor for incident myocardial infarction in the INTERHEART study is also notable, and an important consideration given the intense psychological stress astronauts will experience on long duration space missions (91). Finally, an additional requirement that appears to be lacking across all evaluated models is the inclusion of uncertainty estimates on output measures-this will be needed in the future. The space radiation risk model combines several different sources of both human and animal data for the development of a more comprehensive model that includes several different types of uncertainties. To be consistent with long-term health risks like cancer, a probabilistic risk framework for RICVD is needed where these uncertainties can be quantitatively addressed (92). Ideally, this will include statistical uncertainty associated with CPM calibration but also an estimate on accuracy for the specific target population (astronauts) derived from external validation studies (93).

**Table 4:** Demographic characteristics of the Astro-CHARM/Multimodality risk tool cohorts and astronauts/cosmonauts (21, 22, 89).

**Parameter**	**MESA**	**DHS**	**PACC**	**FHS**	**Astronauts**	**Cosmonauts**
Number	4,029	1,491	1,862	2,057	360	262
Age^*^	55.1 (6.1)	49.8 (6.7)	43.0 (2.6)	49.8 (6.7)	34.4 (3.6)	31.3 (5.4)
Male	1,894 (47%)	656 (44%)	1,527 (82%)	998 (48.5%)	310 (86%)	244 (93%)
Ethnicity						
White	1,491 (37%)	552 (37%)	1,285 (69%)	2,057 (100%)	324 (90%)	258 (98%)
Black	1,088 (27%)	716 (48%)	372 (20%)	0 (0%)	19 (5%)	0 (0%)
Hispanic	967 (24%)	194 (13%)	112 (6%)	0 (0%)	12 (3%)	0 (0%)
Smoking	685 (17%)	417 (28%)	130 (7%)	288 (14%)	N/A	N/A
Diabetes	402 (10%)	164 (11%)	19 (1%)	99 (4.8%)	0 (0%) ^**^	0 (0%) ^**^

* *For astronauts and cosmonauts, the age listed is the age at selection. The data is presented as mean values (standard deviation). MESA, Multi-Ethnic Study of Atherosclerosis; DHS, Dallas Heart Study; PACC, Prospective Army Coronary Calcium Project; FHS, Framingham Heart Study*.

** *Diabetes is a disqualifying condition for astronaut selection*.

### Radiation Risk Modeling

Future improvements in radiation epidemiology will also provide added refinements to NASA's risk assessment models. For example, the Million Worker Study (94) and other large population studies like the International Nuclear Workers Study (95) may provide data that will reduce some of the uncertainty associated with risk transfer from the atomic bomb cohorts (8). These studies include chronically exposed individuals which may be more like exposures in spaceflight, although the dose ranges are low relative to those expected for long-duration missions, and the types of radiation exposure are also quite distinct. Unfortunately, these datasets also lack lifestyle and environmental information such as smoking, diabetes, high blood pressure, high LDL cholesterol, and obesity. Indeed, this is a problem for many radiation epidemiologic datasets with the significant exceptions of the Japanese Atomic Bomb Survivor Life Span Study cohort and the Mayak Worker cohort (96).

It will also be important to acquire more knowledge on the interaction of radiation with traditional CVD risk factors (8, 97). For example, the combined risk from radiation exposure and the lifestyle factors for CVD (e.g., diabetes, smoking, obesity, high blood pressure, elevated total cholesterol and elevated LDL cholesterol) not well understood. Questions remain about the type (e.g., additive, multiplicative, synergistic) and the magnitude of interaction. To investigate this, cohorts exposed to radiation will need further evaluation to identify and characterize the dependence of the radiation risks on these factors. Because of potential limitations in the available information from these cohorts, radiobiology experiments with animal models are needed to evaluate mechanisms and inform the nature of these interactions.

Other space environment factors, such as microgravity and sleep deficiency, also have adverse effects on the circulatory system. It will therefore be necessary to evaluate possible synergistic or antagonistic effects of these non-radiation space environment factors in RICVD risk assessment (16, 98).

Another important consideration is how to bridge the gap between the known sex differences in CVD risk that are considered in the clinical risk models and the possible sex differences in response of the cardiovascular system to radiation exposure. This is an area actively being pursued in ground-based radiobiology research, and is especially important considering the equal inclusion of female astronauts on the Artemis crew who will be the NASA explorers returning to the Moon in this decade (99, 100). Additionally, there are many open questions regarding the biology of space radiation and how exposure during spaceflight will impact the types, latency, and severity of disease of the heart and vasculature. This information is required to accurately perform the risk assessment and management for these missions (13). The use of the adverse outcome pathway approach provides a good way to organize such information (101).

### Non-traditional Risk Factors

As new CVD risk factors are identified, it will be important to evaluate their suitability for inclusion in the risk framework since continuous refinements will support the goal of a truly personalized approach to risk assessment. Several new studies show that non-traditional risk factors or biomarkers could be used for CVD risk calculations. For example, higher resting heart rate was shown to be independently associated with increased risks of all-cause and cardiovascular mortality (102). This is interesting because resting heart rate measurements can be obtained by consumer wearables and are readily available.

Another potential risk factor gaining attention is based on somatic mutations in hematopoietic stem cells, known as clonal hematopoiesis of indeterminate potential (CHIP). CHIP refers to the presence of clonally expanded mutant cells that are found in the bone marrow or the blood in otherwise healthy individuals. CHIP carrier status increases with age, with ~10% of individuals exhibiting these clones by age 50. While the level of risk is dependent on the number of mutant clones, the specific mutations involved, and existence of other risk factors, the presence of CHIP is estimated to almost double the CVD risk and thus has potential to be an important variable as astronauts age (103, 104).

### Future Approaches

Advances in genomic sciences, artificial intelligence, and machine learning have fueled development of personalized medical approaches that will be important considerations for radiation risk modeling as these technologies mature. The strong association of family history with CVD supports the role of genetic predisposition as a risk predictor. Recently, genetic variants associated with CVD outcomes have been identified through genome wide association studies. This body of work has provided information on genetic drivers of disease, novel insights into the biology of CVD, and potential treatment opportunities (105). These studies have spurred development of polygenic risk scores that can provide lifetime estimates of risk and may also add to the discriminative ability of clinical risk factors (106), thereby improving risk prediction outside traditional factors (107). The advantages include earlier diagnosis of potential risk (allowing time for adjustment of lifestyle factors), early treatments, and the ability to tailor pharmacologic interventions in a personalized fashion.

Understanding how radiation interacts with the genetic drivers of CVD and also understanding how genetic and epigenetic factors control individual susceptibility to radiation will be important pieces of this complex story (108, 109). Flight surgeons and medical professionals can use this information in private, clinical practice. The Genetic *Information Nondiscrimination* Act of 2008 would prevent the use of such information for some applications, and NASA does not use genetic information for certain decisions such as flight assignment. As this field advances, the use of polygenic risk data and information on individual susceptibilities could be very informative for guiding personal, primary CVD prevention strategies (110).

CPMs that utilize machine learning are another alternative to commonly used statistical approaches. Machine learning is potentially relevant to estimation of risk, even in a small population. The models (both radiation and background risks) are developed from much larger non-astronaut populations. The critical issue is one of validating the model predictions in relatively healthy populations with similar characteristics to the astronauts. Most standard CVD CPMs make an implicit assumption that each risk factor is related in a simple fashion to CVD outcomes. Such models may thus oversimplify complex relationships that include large numbers of risk factors with non-linear interactions (110). Neural networks were shown to be better able to interpret data compared to current methods, helping to provide more precise risk stratification for individuals (111).

Non-invasive imaging approaches may be especially useful for monitoring disease development during long-duration space missions. For example, deep learning neural networks have been used to identify individuals at high risk for CVD by evaluating retinal blood vessel images (112). However, since these approaches are new, significant validation and confirmation work is needed before they can be included in a risk model suitable for NASA use.

In summary, significant advances in the assessment and communication of health risks in the clinic provide opportunities for improvement in the evaluation and management of space radiation risks for astronauts. The dual-use framework described here provides a strategy for individualizing radiation risk assessment for spaceflight operational management, and at the individual level, to guide clinical decisions regarding appropriate CVD surveillance and prevention strategies. This approach is a step toward the longer-term goal of personalizing both risk assessment and treatment options that will ensure astronaut health and safety for space exploration missions.

## Author Contributions

JH, IP, ZP, and RN wrote the manuscript. SB, ML, AK, and LS contributed to editing the manuscript. All authors contributed to the article and approved the submitted version.

## Funding

This work was supported by the Human Research Program of the Human Exploration and Operations Mission Directorate of the National Aeronautics and Space Administration [JH, SB, RN, and LS] and by the Human Health and Performance contract NNJ15HK11B [IP and ZP]; by the Intramural Research Program of the National Institutes of Health, National Cancer Institute, Division of Cancer Epidemiology and Genetics [ML]; NASA 80NSSC19M0207 [AK].

## Author Disclaimer

The opinions expressed in this work are the author's own and do not reflect the view of the National Institutes of Health, the Department of Health and Human Services, or the United States government.

## Conflict of Interest

Several authors work directly (JH, SB, RN, and LS) as employees or indirectly as contractors (IP and ZP) for NASA, the views and opinions expressed herein are those of the authors and do not necessarily reflect the views of NASA or the United States government and do not imply NASA supported program direction. IP and ZP were employed by KBR. The remaining authors declare that the research was conducted in the absence of any commercial or financial relationships that could be construed as a potential conflict of interest.

## Publisher's Note

All claims expressed in this article are solely those of the authors and do not necessarily represent those of their affiliated organizations, or those of the publisher, the editors and the reviewers. Any product that may be evaluated in this article, or claim that may be made by its manufacturer, is not guaranteed or endorsed by the publisher.

## Abbreviations

ASCVD: atherosclerotic cardiovascular disease
CHIP: clonal hematopoiesis of indeterminate potential
CPM: clinical prediction model
CVD: cardiovascular disease
GCR: galactic cosmic ray
REID: risk of exposure-induced death
RICVD: radiation-induced cardiovascular disease.
