# Supplementary material for: Cardiovascular Disease Risk Modeling for Astronauts: Making the Leap From Earth to Space
Source: Front Cardiovasc Med. 2022 May 19;9:873597. doi: 10.3389/fcvm.2022.873597 (PMC9161032; doi:10.3389/fcvm.2022.873597)
Supplement: Supplementary file 1 [file Data_Sheet_1.pdf]

Supplementary Material

6. Survey of CVD CPMs (cont'd)

6.3 Framingham Risk Score

The Framingham Heart Study is a long-term, ongoing cardiovascular cohort study that first started in Framingham, Massachusetts in 1948. Several cohorts have been added to the original group: 1971 (children), 1994 (Omni (multiethnic)), and 2002 (third generation). In D'Agostino et al. (2008) (1), the Framingham Heart Study investigators developed a 10-year general cardiovascular disease (CVD) clinical prediction model (CPM), the Framingham Risk Score, with C-statistics ranging from 0.746-0.780. This model predicts a composite of coronary heart disease (coronary death, myocardial infarction, coronary insufficiency, and angina), cerebrovascular events and transient ischemic attacks as well as peripheral artery disease and heart failure under the umbrella term cardiovascular disease. 30-year risk prediction functions were developed from a subset of the Framingham Offspring cohort who were free from CVD and with ages 20 to 59 and at the start of the study. Hard CVD event endpoints (coronary death, myocardial infarction, stroke) were evaluated. The C-statistic of this predictor was 0.803 and standard risk factors were evaluated (2). A main limitation is that the Framingham Heart Study cohort is predominantly Caucasian from a restricted geographic area and is not necessarily representative of the total United States population.

6.4 ACC/AHA PCE ASCVD Risk Estimator & ASCVD Risk Estimator Plus

In 2013, the American College of Cardiology (ACC), the American Heart Association (AHA), and the National Heart, Lung, and Blood Institute collaborated to develop clinical practice guidelines for assessment of CVD risk, including development of a new CVD CPM (3,4). This tool uses comprehensive multivariable risk equations, the Pooled Cohort Equations, for the prediction of 10-year risk of atherosclerotic CVD (ASCVD; fatal and non-fatal myocardial infarction and stroke) in non-Hispanic African American and non-Hispanic White men and women from 40 to 79 years of age. The C-statistics reported for this model are 0.713-0.818. This CPM is currently the most widely used in US clinical practice for preventive therapies, including cholesterol management. However, some studies have suggested it may overestimate risk by as much as 50% (5). The ASCVD Risk Estimator Plus, builds on the original ASCVD Risk Estimator with addition of the 2016 Million Hearts Longitudinal ASCVD Risk Assessment Tool, to predict benefits from inclusion of specific primary preventive strategies (aspirin, blood pressure control, cholesterol lowering medications and smoking cessation). Recommendations for estimating lifetime or 30-yr ASCVD risk are included (2,6).

6.5 MESA Risk Score

The Multi-Ethnic Study of Atherosclerosis (MESA) risk score was the first to incorporate coronary artery calcium data as a marker of subclinical CVD, in addition to traditional risk factors, to provide a 10-year coronary heart disease risk estimate (7). Study participants were 6814 healthy female and male individuals from the MESA cohort ranging in age from 45 to 84. Like the Framingham risk score, the MESA model predicts hard coronary heart disease endpoints (myocardial infarction, resuscitated cardiac arrest, fatal coronary heart disease and revascularization). The C-statistic for the model is 0.80. In comparison, both the Framingham and the 2013 ACC/AHA calculators had lower C-statistics, ranging from 0.65-0.75 when evaluated using MESA cohort data. The MESA risk score also showed superior performance when compared to use of the existing 2013 AHA/ACC score with coronary artery calcium added in, where the C-statistic was 0.78. The use of a multi-ethnic cohort for model development is an advantage of this calculator, and inclusion of coronary artery calcium provides improved model discrimination.

6.6 Reynolds Risk Score

The Reynolds Risk Score was developed to specifically address the need for more accurate risk calculators tailored to women. This model provides a global or absolute assessment for a 10-year risk of the first cardiovascular event comprising myocardial infarction, ischemic stroke, coronary revascularization, and cardiovascular death (8). In the initial study, 35 factors were assessed for CVD risk in 24,558 healthy women enrolled in the Women’s Health Study using Bayesian information criteria. Study participants were age 45 and older with no history of CVD. Following this analysis, 9 risk factors were selected and used to create model A. These factors are sex, age, smoking, systolic blood pressure, total cholesterol, high-density lipoprotein cholesterol, high-sensitivity C-reactive protein, and parental history of premature disease, which is a strong predictor of individual risk. The C-statistics for this model is 0.81. Once variables were selected from model A, a second, simpler CPM (model B, the Reynolds Risk Score) was created for the purpose of clinical application and efficiency. This model uses parental history and high-sensitivity C-reactive protein; risk classification is based on these values: <5%, 5-10%, 10-20%, >20%. A similar calculator incorporating high-sensitivity C-reactive protein and parental family history was developed for men (9). Here participants (10,724 healthy males over age 50) were part of the Physician’s Health Study II. The addition of high-sensitivity C-reactive protein and parental history were shown to improve global risk prediction (9). Derivation from a predominantly white study population may be a limiting factor, while the inclusion of high-sensitivity C-reactive protein is notable as this biomarker is currently evaluated in astronauts (10).

6.7 INTERHEART Modifiable Risk Score

In this work, the CPMs (short score and full score) are based on a case-control study of acute myocardial infarction in 52 countries, with the outcome of interest being the first acute myocardial infarction event (11,12). The data was examined using a logistic regression model. The risk factors

considered in this study are age, sex, dyslipidemia, smoking, hypertension, diabetes, abdominal obesity, psychosocial factors, consumption of fruits and vegetables, alcohol use, and regular physical activity. The overall C-statistics for this model is 0.70 – 0.72 and increases modestly when adding variables (0.72-0.74), in some areas of the world. The score has been validated using an independent study population for a 3.25-year follow-up. Psychosocial stress assessment consisted of an integrated score that considered depression, locus of control, perceived stress, and life events. Identification of psychosocial stress as an independent risk factor for acute myocardial infarction is especially relevant for astronauts considering the psychological stress associated with isolation and confinement on long duration space missions (13).

6.8 SCORE / SCORE2

The European Cardiovascular Disease Risk Assessment Model (SCORE) was developed by the European Society of Cardiology in 2003 and updated in 2016 (14,15). It includes 12 European cohort studies comprising a total of 205,178 persons (88,080 women and 117,098 men) with 2.7 million person-years of follow-up. Calculators were developed for the 10-year risk of fatal CVD, which includes separate equations for coronary heart disease and non-coronary CVD. Different calculators are used for low-risk and high-risk regions of Europe. The model is based on fatal events only (non-fatal events are dependent on definitions and methods). The risk functions underlying the risk charts were calculated using a Weibull proportional hazards model. The risk predictors are age, cholesterol, smoking, systolic blood pressure, diabetes, total cholesterol, and high-density lipoprotein cholesterol. The C-statistics for this model are 0.71-0.84. SCORE2 updates were published in 2021 and incorporate non-fatal CVD risk (defined as non-fatal myocardial infarction, non-fatal stroke) using updated information from 45 cohorts in 13 countries (677,684 individuals). The model was recalibrated to accommodate 4 risk regions in Europe; C-statistics are 0.67 to 0.81 (16). Given the development and validation in European cohorts, SCORE CPMs may have less generalizability to United States-based astronauts.

6.9 QRISK2 / QRISK3

The first version of this QRISK calculator was published in 2007, updated in 2008 (QRISK2), and again most recently in 2017 (QRISK3) (17–19). This calculator evaluates 10-year and lifetime risks of CVD (coronary heart disease, ischemic stroke, and transient ischemic attack). Cox proportional hazards models were used to estimate the coefficients for each risk factor in women and men from the United Kingdom separately. This calculator uses the largest number of predictor variables among the surveyed CPMs: age, ethnicity, deprivation, systolic blood pressure, body mass index, total cholesterol, high-density lipoprotein: cholesterol ratio, smoking, family history of coronary heart disease in a first degree relative aged less than 60 years, diabetes, treated hypertension, rheumatoid arthritis, atrial fibrillation, and chronic kidney disease. The latest version adds additional new risk factors including chronic kidney disease, systolic blood pressure variability, migraines, use of corticosteroids, systemic lupus erythematosus, use of atypical

antipsychotics and severe mental illness. The derivation cohort included data from 7.89 million patients aged 25-84 years and 2.67 million patients in the validation cohort (derived from the QResearch database of 30 million patients). The C-statistic for this model is 0.88. A version of the model was also developed to predict cumulative risk measured over a full lifetime to address the needs of risk estimation in younger individuals – its C-statistic was 0.84 for women and 0.83 for men (20). These models lack inclusion of biomarkers and their performance in United States populations is unclear.

6.10 LIFE-CVD model

The LIFEtime-perspective CardioVascular Disease (LIFE-CVD) model aims to individualize CVD prevention strategies in asymptomatic individuals (21,22). The model used 6715 participants from the MESA cohort. The model estimates the effects of antithrombotic therapy, blood pressure lowering, smoking cessation and cholesterol management on 10-year and lifetime CVD risk (defined as fatal or non-fatal MI, stroke, resuscitated cardiac arrest and death due to coronary heart disease). The model also outputs CVD-free life expectancy. Covariates are sex, systolic blood pressure, non-high-density lipoprotein cholesterol, body mass index, smoking status (current, former, never), diabetes, family history of early MI in either parent. The C-statistic is 0.74. An advantage of this model is that it uses an approach similar to the NASA radiation risk model, outputting results by year.

References

1. D'Agostino RB, Vasan RS, Pencina MJ, Wolf PA, Cobain M, Massaro JM, Kannel WB. General cardiovascular risk profile for use in primary care: the Framingham Heart Study. *Circulation* (2008) **117**:743–753. doi: 10.1161/CIRCULATIONAHA.107.699579

2. Pencina MJ, D'Agostino RB, Larson MG, Massaro JM, Vasan RS. Predicting the Thirty-year Risk of Cardiovascular Disease: The Framingham Heart Study. *Circulation* (2009) **119**:3078–3084. doi: 10.1161/CIRCULATIONAHA.108.816694

3. Lin JS, Evans CV, Johnson E, Redmond N, Coppola EL, Smith N. Nontraditional risk factors in cardiovascular disease risk assessment: Updated evidence report and systematic review for the US Preventive Services Task Force. *JAMA* (2018) **320**:281–297. doi: 10.1001/jama.2018.4242

4. Goff DC, Lloyd-Jones DM, Bennett G, Coady S, D'Agostino RB, Gibbons R, Greenland P, Lackland DT, Levy D, O'Donnell CJ, et al. 2013 ACC/AHA guideline on the assessment of cardiovascular risk: a report of the American College of Cardiology/American Heart Association Task Force on Practice Guidelines. *Circulation* (2014) **129**:S49-73. doi: 10.1161/01.cir.0000437741.48606.98

5. DeFilippis AP, Young R, McEvoy JW, Michos ED, Sandfort V, Kronmal RA, McClelland RL, Blaha MJ. Risk score overestimation: the impact of individual cardiovascular risk factors and preventive therapies on the performance of the American Heart Association-American

College of Cardiology-Atherosclerotic Cardiovascular Disease risk score in a modern multi-ethnic cohort. *Eur Heart J* (2017) **38**:598–608. doi: 10.1093/eurheartj/ehw301

6. Berry JD, Dyer A, Cai X, Garside DB, Ning H, Thomas A, Greenland P, Van Horn L, Tracy RP, Lloyd-Jones DM. Lifetime Risks of Cardiovascular Disease. *N Engl J Med* (2012) **366**:321–329. doi: 10.1056/NEJMoa1012848

7. McClelland RL, Jorgensen NW, Budoff M, Blaha MJ, Post WS, Kronmal RA, Bild DE, Shea S, Liu K, Watson KE, et al. 10-Year Coronary Heart Disease Risk Prediction Using Coronary Artery Calcium and Traditional Risk Factors: Derivation in the MESA (Multi-Ethnic Study of Atherosclerosis) With Validation in the HNR (Heinz Nixdorf Recall) Study and the DHS (Dallas Heart Study). *J Am Coll Cardiol* (2015) **66**:1643–1653. doi: 10.1016/j.jacc.2015.08.035

8. Ridker PM, Buring JE, Rifai N, Cook NR. Development and validation of improved algorithms for the assessment of global cardiovascular risk in women: The Reynolds Risk Score. *JAMA* (2007) **297**:611–619. doi: 10.1001/jama.297.6.611

9. Ridker PM, Paynter NP, Rifai N, Gaziano JM, Cook NR. C-reactive protein and parental history improve global cardiovascular risk prediction: The Reynolds Risk Score for men. *Circulation* (2008) **118**:2243–2251. doi: 10.1161/CIRCULATIONAHA.108.814251

10. Lee SMC, Ribeiro LC, Martin DS, Zwart SR, Feiveson AH, Laurie SS, Macias BR, Crucian BE, Krieger S, Weber D, et al. Arterial structure and function during and after long-duration spaceflight. *J Appl Physiol Bethesda Md 1985* (2020) **129**:108–123. doi: 10.1152/japplphysiol.00550.2019

11. McGorrian C, Yusuf S, Islam S, Jung H, Rangarajan S, Avezum A, Prabhakaran D, Almahmeed W, Rumboldt Z, Budaj A, et al. Estimating modifiable coronary heart disease risk in multiple regions of the world: the INTERHEART Modifiable Risk Score. *Eur Heart J* (2011) **32**:581–589. doi: 10.1093/eurheartj/ehq448

12. Yusuf S, Hawken S, Ôunpuu S, Dans T, Avezum A, Lanas F, McQueen M, Budaj A, Pais P, Varigos J, et al. Effect of potentially modifiable risk factors associated with myocardial infarction in 52 countries (the INTERHEART study): Case-control study. *The Lancet* (2004) **364**:937–952. doi: 10.1016/S0140-6736(04)17018-9

13. Pagel JI, Choukèr A. Effects of isolation and confinement on humans-implications for manned space explorations. *J Appl Physiol Bethesda Md 1985* (2016) **120**:1449–1457. doi: 10.1152/japplphysiol.00928.2015

14. Conroy RM, Pyörälä K, Fitzgerald AP, Sans S, Menotti A, De Backer G, De Bacquer D, Ducimetière P, Jousilahti P, Keil U, et al. Estimation of ten-year risk of fatal cardiovascular disease in Europe: the SCORE project. *Eur Heart J* (2003) **24**:987–1003. doi: 10.1016/s0195-668x(03)00114-3

15. Piepoli MF, Hoes AW, Agewall S, Albus C, Brotons C, Catapano AL, Cooney M-T, Corrà U, Cosyns B, Deaton C, et al. 2016 European Guidelines on cardiovascular disease

prevention in clinical practice: The Sixth Joint Task Force of the European Society of Cardiology and Other Societies on Cardiovascular Disease Prevention in Clinical Practice (constituted by representatives of 10 societies and by invited experts). Developed with the special contribution of the European Association for Cardiovascular Prevention & Rehabilitation (EACPR). *Eur Heart J* (2016) **37**:2315–2381. doi: 10.1093/eurheartj/ehw106

16. SCORE2 working group and ESC Cardiovascular risk collaboration. SCORE2 risk prediction algorithms: new models to estimate 10-year risk of cardiovascular disease in Europe. *Eur Heart J* (2021) **42**:2439–2454. doi: 10.1093/eurheartj/ehab309

17. Hippisley-Cox J, Coupland C, Vinogradova Y, Robson J, May M, Brindle P. Derivation and validation of QRISK, a new cardiovascular disease risk score for the United Kingdom: prospective open cohort study. *BMJ* (2007) **335**:136. doi: 10.1136/bmj.39261.471806.55

18. Hippisley-Cox J, Coupland C, Vinogradova Y, Robson J, Minhas R, Sheikh A, Brindle P. Predicting cardiovascular risk in England and Wales: prospective derivation and validation of QRISK2. *BMJ* (2008) **336**:1475–1482. doi: 10.1136/bmj.39609.449676.25

19. Hippisley-Cox J, Coupland C, Brindle P. Development and validation of QRISK3 risk prediction algorithms to estimate future risk of cardiovascular disease: prospective cohort study. *BMJ* (2017) **357**:j2099. doi: 10.1136/bmj.j2099

20. Hippisley-Cox J, Coupland C, Robson J, Brindle P. Derivation, validation, and evaluation of a new QRISK model to estimate lifetime risk of cardiovascular disease: cohort study using QResearch database. *BMJ* (2010) **341**:c6624–c6624. doi: 10.1136/bmj.c6624

21. Jaspers NEM, Blaha MJ, Matsushita K, van der Schouw YT, Wareham NJ, Khaw K-T, Geisel MH, Lehmann N, Erbel R, Jöckel K-H, et al. Prediction of individualized lifetime benefit from cholesterol lowering, blood pressure lowering, antithrombotic therapy, and smoking cessation in apparently healthy people. *Eur Heart J* (2020) **41**:1190–1199. doi: 10.1093/eurheartj/ehz239

22. de Vries TI, Jaspers NEM, Visseren FLJ, Dorresteijn JAN. An update to the LIFEtime-perspective CardioVascular Disease (LIFE-CVD) model for prediction of individualized lifetime benefit from cardiovascular risk factor management in apparently healthy people. *medRxiv* (2021)2021.03.15.21253400. doi: 10.1101/2021.03.15.21253400
